# New Insights in Hydrogels for Periodontal Regeneration

**DOI:** 10.3390/jfb14110545

**Published:** 2023-11-11

**Authors:** Mafalda S. Santos, Alexandra B. dos Santos, Marta S. Carvalho

**Affiliations:** 1 Department of Bioengineering, iBB—Institute for Bioengineering and Biosciences, Instituto Superior Técnico, Universidade de Lisboa, 1049-001 Lisboa, Portugal; mafaldasantos4@tecnico.ulisboa.pt (M.S.S.); alexandrabettencourt@tecnico.ulisboa.pt (A.B.d.S.); 2 Associate Laboratory i4HB—Institute for Health and Bioeconomy at Instituto Superior Técnico, Universidade de Lisboa, 1049-001 Lisboa, Portugal

**Keywords:** alveolar bone, cementum, hydrogels, periodontal ligament, periodontitis

## Abstract

Periodontitis is a destructive inflammatory disease characterized by microbial infection that damages the tissues supporting the tooth (alveolar bone, gingiva, periodontal ligament, and cementum), ultimately resulting in the loss of teeth. The ultimate goal of periodontal therapy is to achieve the regeneration of all of the periodontal tissues. Thus, tissue engineering approaches have been evolving from simple membranes or grafts to more complex constructs. Hydrogels are highly hydrophilic polymeric networks with the ability to simulate the natural microenvironment of cells. In particular, hydrogels offer several advantages when compared to other forms of scaffolds, such as tissue mimicry and sustained drug delivery. Moreover, hydrogels can maintain a moist environment similar to the oral cavity. Hydrogels allow for precise placement and retention of regenerative materials at the defect site, minimizing the potential for off-target effects and ensuring that the treatment is focused on the specific defect site. As a mechanism of action, the sustained release of drugs presented by hydrogels allows for control of the disease by reducing the inflammation and attracting host cells to the defect site. Several therapeutic agents, such as antibiotics, anti-inflammatory and osteogenic drugs, have been loaded into hydrogels, presenting effective benefits in periodontal health and allowing for sustained drug release. This review discusses the causes and consequences of periodontal disease, as well as the advantages and limitations of current treatments applied in clinics. The main components of hydrogels for periodontal regeneration are discussed focusing on their different characteristics, outcomes, and strategies for drug delivery. Novel methods for the fabrication of hydrogels are highlighted, and clinical studies regarding the periodontal applications of hydrogels are reviewed. Finally, limitations in current research are discussed, and potential future directions are proposed.

## 1. Introduction

**Figure 1:**
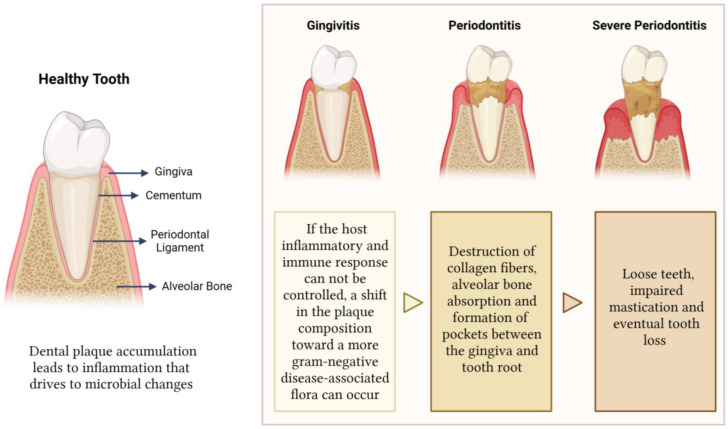
The structure of periodontium and the different stages of periodontal disease. It first manifests as gingivitis and then progresses to a more serious infection affecting the soft tissue and the alveolar bone that support the teeth. If left untreated, it can result in tooth loss. Figure created using Biorender.com.

Periodontal disease is a multifactorial disease in which pathogenic bacteria initiate the host immune response, leading to the destruction of the tissues surrounding and supporting the teeth, such as gingiva, periodontal ligament, alveolar bone, and cementum [1]. These four tissues are part of the periodontium, a supporting apparatus essential for the restoration and proper functioning of periodontal tissues (Figure 1) [2]. Apart from anchoring the teeth in their respective alveolar pockets, the periodontium also stabilizes them by distributing and absorbing masticatory forces, and it serves as a barrier against several pathogens [2]. Periodontal disease first manifests as gingivitis, a dental inflammation initiated by dental plaque accumulation and affected by the host response, which dictates the disease progression [3]. Although improvements in oral hygiene habits may reverse gingivitis, a lack of treatment can lead to periodontitis, which is characterized by the destruction of collagen fibers, alveolar bone absorption, and the formation of soft tissue pockets between the gingiva and tooth root. If not addressed properly, this disease can progress to severe periodontitis, causing loose teeth, impaired mastication, and eventual tooth loss [1] (Figure 1). The loss of teeth due to severe periodontitis may lead to alveolar bone atrophy, which may require important bone augmentation or a bone graft in order to perform correct implant-supported prosthodontics [4].

Periodontitis is a public health problem, and its most severe form is the eleventh-most prevalent human disease [5]. Periodontitis affects 20% to 50% of the worldwide population, and its severity can be associated with sociodemographic variables, such as age and income [6]. In fact, different age groups are disproportionally affected by the disease, since the prevalence and severity of periodontitis tend to increase with age [5]. Furthermore, Nazir and colleagues showed that low-income subjects had significantly higher chances of having severe periodontal disease than high-income subjects [5]. In addition, periodontal disease represents a risk factor for surgical infection after mandibular fracture and for mandibular fracture [7].

Because it is caused by bacterial inflammation and plaque accumulation, clinical treatments of periodontitis focus on cause-related, non-surgical, and conservative approaches, such as plaque removal and local inflammation control [8]. Thus, the first clinical strategy is usually a debridement treatment involving mechanically cleaning the periodontal pockets to remove bacteria. However, if deep pockets are present, resective surgery might also be needed [9]. Although these therapies minimize symptoms and prevent further disease progression, they are not able to restore all the lost tissues, including the periodontal ligament, leaving patients with functional and aesthetic sequelae [10]. Aiming to surpass the limitations of the current treatments and to improve the outcomes of standard therapy, tissue engineering strategies have been explored for periodontal regeneration.

The biology of periodontal regeneration involves a complex interplay between various elements, including cells (fibroblasts, osteogenic cells, and immune cells), bioactive molecules (growth factors), and the extracellular matrix (ECM) [11]. The interaction between these components is highly orchestrated. Following injury or disease, cells respond to signals from the local environment and bioactive molecules. They then produce the necessary ECM components to create new tissue. Growth factors and other signaling molecules guide the cells’ behavior, influencing their differentiation and function [12] (Table 1). The whole process faces several biological and clinical challenges, such as its spatiotemporal healing coordination, the competition between tissues, and the clinically challenging surgical environment [13]. In fact, to achieve complete regeneration, it is necessary to reconstruct the whole periodontium, including the alveolar bone and new cementum, with the insertion of the functionally oriented collagen fibers of a newly formed periodontal ligament [2,14].

In periodontal regenerative therapies, several scaffold forms have been used to support tissue regeneration and healing, including membranes, sponges, fibers, 3D-printed scaffolds, and hydrogels. In particular, hydrogels offer several advantages compared to other forms of scaffolds, such as tissue mimicry and sustained drug delivery. Hydrogels are highly hydrophilic polymeric networks with the ability to simulate the natural microenvironment of cells, and they can be developed from natural or synthetic polymers [15]. Moreover, hydrogels can maintain a moist environment that is similar to the oral cavity. Hydrogels allow for the precise placement and retention of regenerative materials at the site of interest [16]. This minimizes the potential for off-target effects and ensures that the treatment is focused on the specific defect site.

Hydrogels derived from natural polymers, including alginate, cellulose, chitosan, collagen, fibrin, gelatin, and hyaluronic acid, have been used in several biomedical applications, such as 3D cell cultures, drug delivery systems, wound healing, and tissue regeneration [17,18]. Natural hydrogels are biocompatible, bioactive, they present low cytotoxicity, and their structure resembles native tissues [19,20]. However, hydrogels do not have strong mechanical properties and they present batch-to-batch variability in composition, impairing the tuning of the material properties [21]. Natural hydrogels often rely on proteins or polysaccharides with batch-specific variations in their characteristics, such as molecular weight, charge, and structural integrity. These variations can affect the overall properties of the hydrogel, including its mechanical strength, swelling capacity, and biocompatibility. On the other hand, synthetic hydrogels, such as polyvinyl alcohol (PVA), polyethylene glycol (PEG) [22], polyacrylic acid (PAA), or polyacrylamide (PAAM), do not present these disadvantages; however, they lack the endogenous factors that are required to promote cell behavior, such as migration, proliferation, and differentiation [21]. Different techniques can be used to fabricate hydrogels tailored to specific applications with the required chemical and physical properties [18]. This review covers the current available treatments of periodontal disease, describing the use of bone graft materials, guided tissue regeneration, and enamel matrix derivatives. Alternative tissue engineering strategies are explored, in particular the use of 3D hydrogels. The recent research on 3D hydrogels for periodontal regeneration is summarized and discussed. Novel methods for the fabrication of hydrogels are highlighted, and clinical studies regarding the application of hydrogels for periodontal applications are reviewed. Finally, the shortcomings and future perspectives of using 3D hydrogels for treatment of periodontitis are discussed.

## 2. Current Treatments

Periodontal therapy aims to regenerate all the tissues damaged due to periodontal disease. The regeneration of these tissues that compose the periodontium involves distinct cell types, including periodontal ligament stem/stromal cells (PDLSC), alveolar bone cells, cementoblasts, and epithelial cells [23,24]. Among the current treatments, guided tissue regeneration (GTR) membranes and bone grafts are the most used in clinics [11]. Importantly, the clinical image of the defect site in clinical settings plays a critical role in optimizing the probability of successful regeneration. However, these approaches present a lack of compartmentalization between the periodontal defect and the surrounding soft tissue, leading to poor regenerative outcomes. Hence, several strategies have been introduced to regenerate the whole periodontium, such as the use of enamel matrix derivatives (EMD) and other growth factors, as well as tissue engineering strategies, including 3D hydrogels.

### 2.1. Bone Graft Materials

**Figure 2:**
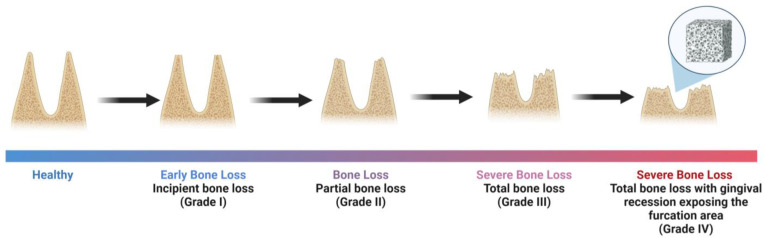
Bone loss triggered by periodontal disease. Bone grafts can be used to enhance the alveolar bone to accommodate an implant or to preserve the natural teeth in that defect, promoting new bone formation. Figure created using Biorender.com.

Bone graft materials have been used to fill in periodontal defects, promoting alveolar bone regeneration (Figure 2). To achieve bone regeneration, the residual bony walls of the periodontal defect need to be able to provide mechanical support and blood supply to the bone graft [25]. Bone grafts have a supportive function and should present osteogenic, osteoconductive, and osteoinductive properties to allow for new bone formation as well as bioactive sites for cells to proliferate while recruiting host stem cells into the defect site [25,26].

Different types of grafts have been used, such as autologous, allogeneic, and xenogeneic grafts [27]. Furthermore, considering the material, grafts can be further divided into five distinct categories: natural, synthetic, composite, growth factor-based bone substitutes, and bone substitutes with infused living cells [26]. Natural bone grafts represent the majority of bone grafts used worldwide, and include autografts, allografts (such as demineralized bone matrix and freeze-dried bone matrix), xenografts (such as chitosan or silk from other species), and plant-based materials (such as algae- and coral-based grafts) [26,28] (Table 2). Nevertheless, natural bone grafts present several disadvantages. In fact, although autografts are considered the gold standard for bone graft materials due to their osteogenic properties and low risk of immunogenicity, the harvest procedure is associated with pain and higher morbidity at the donor site. On the other hand, allografts may trigger an immune response due to graft rejection by the recipient [28,29,30]. Therefore, synthetic bone substitutes aim to mimic the properties of natural bone while tackling the aforementioned limitations [26]. Calcium phosphate ceramics, calcium phosphate cements, metals, and polymers are examples of the synthetic bone substitutes used [26]. Different materials can also be combined in order to achieve an optimal set of mechanical properties, forming composites. Examples of commercialized composites are NanoBone^TM^ (76% *w*/*w* nanocrystalline hydroxyapatite and 24% *w*/*w* silicon) [31] and Fortoss Vital^TM^ (β-tricalcium phosphate (β-TCP) in a calcium sulphate matrix) [32]. Aiming to improve the osteoinductive properties of the grafts, growth factor-based bone substitutes have been used. Platelet-derived growth factor (PDGF), bone morphogenetic proteins (BMP) such as BMP-2 and BMP-7, fibroblast growth factor 2 (FGF-2), parathyroid hormone (PTH), insulin-like growth factors (IGF), and platelet concentrates such as platelet-rich plasma (PRP) and platelet-rich fibrin (PRF) have been used for alveolar bone grafts and substitutes [26,29].

Infuse^TM^ bone graft is an example of a commercialized product for periodontal regeneration. Infuse^TM^ bone graft is approved for use in sinus augmentation and localized alveolar ridge augmentation [40]. It contains recombinant human BMP-2, inducing new bone formation [26].

Finally, bone substitutes with infused living cells have been explored to treat periodontal defects, aiming to increase the osteogenic and osteoconductive properties of the material [26]. Mesenchymal stem/stromal cells (MSC) have been widely used in the dentistry field, since they are able to differentiate towards an osteogenic lineage and are able to regenerate large bone defects when combined with a scaffold [26,41].

Although they are able to regenerate bone tissue, current bone grafts alone are not able to prevent epithelium downgrowth and have also been used in combination with other approaches, such as GTR [42]. In fact, bone grafting procedures have demonstrated the formation of a long junctional epithelium rather than a new connective tissue attachment [27].

### 2.2. Guided Tissue Regeneration

**Figure 3:**
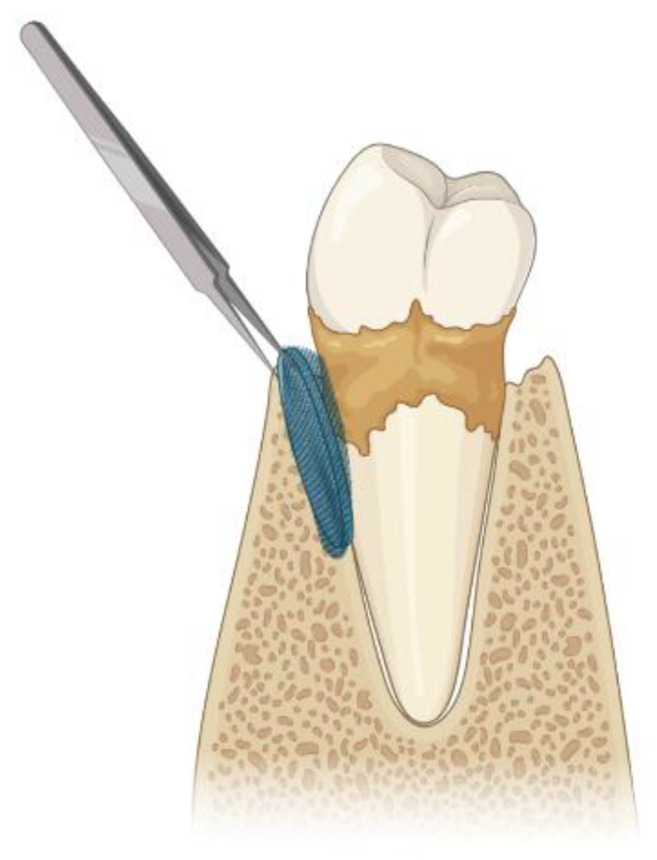
Illustration of the guided tissue regeneration technique used for periodontal therapy. Figure created using Biorender.com.

GTR uses membranes to act as physical barriers, avoiding connective and epithelial tissue downgrowth into the defect [43,44] (Figure 3). Besides excluding epithelial cells from the defect, these membranes also provide space for cells, such as PDLSC, osteoblasts, and cementoblasts, to repopulate the wound area while increasing wound stabilization [29,45].

Although periodontal regeneration using GTR strategies has shown quite satisfactory results in animal models, the same was not observed in clinical settings. The clinical outcomes varied according to the nature of the periodontal defect as well as the skills and experience of the clinician [13]. These poor clinical outcomes are associated with the inability of progenitor cells to repopulate the defects in a certain spatial or temporal order [13]; a very important requirement to achieve multiple tissue regeneration and functional restoration [42].

These membranes should exhibit four critical parameters: biocompatibility, an adequate degradation time matching the rate of new tissue formation, proper mechanical/physical properties, and sufficient sustained strength to avoid membrane collapse [46]. Polytetrafluoroethylene (PTFE) membranes either with or without titanium reinforcement were the gold standard of non-resorbable membranes [23,29]; however, this type of membrane presents several drawbacks. Among them, there is a need for a second surgery for their removal, representing additional costs, as well as pain and discomfort for the patients, negatively affecting regenerative outcomes [23,29,43]. To address these shortcomings, biodegradable membranes were introduced. Indeed, resorbable membranes are used to reduce patient discomfort and to accelerate tissue healing through their bioactive properties [23]. Although natural membranes present good biocompatible and bioactive properties, they also present some disadvantages, such as poor mechanical properties and fast and unpredictable degradation rates [23]. Synthetic resorbable membranes have also been used, since their degradation and mechanical properties can be easily tailored [23]. Polylactic acid (PLA), polyglycolic acid (PGA), and their copolymers are the most used for these purposes [11].

Compared to the use of hydrogels, GTR membranes involve a surgical procedure with potential complications. Furthermore, maintaining the stability and integrity of the barrier membrane during the healing period is challenging. Membrane displacement can compromise the success of the procedure.

### 2.3. Enamel Matrix Derivatives

Recently, EMD have been used to treat periodontal defects. EMD are mainly composed of enamel matrix proteins, 90% of which are amelogenins, and the remaining 10% are prolin-rich non-amelogenins, tuftelin, and other serum proteins [47]. Several studies have reported that EMD can mimic the biological processes that occur during periodontal tissue formation; however, the mechanism of action remains unclear. In fact, EMD are involved in the formation of acellular cementum; the most important tissue for the insertion of collagen fibers [47]. Furthermore, this mixture of proteins plays a significant role in the development of the periodontium [48], since EMD upregulate Runt-related transcription factor 2 (RUNX2) and Osterix (OSX) transcription factors [48] and increase the production of transforming growth factor-β (TGF-β), BMP, vascular endothelial growth factors (VEGF), and FGF-2 [49]. In addition to the stimulatory effects of growth and transcription factors during periodontal wound healing, EMD can retard epithelial downgrowth [49]. Emdogain^®^ (Straumann, Basel, Switzerland) is a commercially available product composed of EMD derived from the developing teeth germs of six-month old piglets combined with a vehicle solution of propylene glycol alginate [8]. Emdogain^®^ is an injectable hydrogel. It is minimally invasive and possesses antimicrobial effects, eliminating the need for antibiotic coverage [9]. However, being a porcine-derived product, Emdogain^®^ might present ethical concerns and trigger an immune response once applied in humans. Even so, Emdogain^®^ has been one of the most used approaches in clinics, in part due to EMD being quite similar among mammalian species, which translates in a smaller antigenic potential [9,30]. Regarding expected outcomes, in specific cases, this treatment is effective for the regeneration of alveolar bone, cementum, and periodontal ligament [50]. Although several studies have reported the capacity of EMD to promote the regeneration of periodontal tissues as well as to improve the clinical attachment levels and reduce probing pocket depth [9,49,51], a high degree of heterogeneity among the results has also been shown [9]. Moreover, due to its gel-like consistency, Emdogain^®^ has been used in combination with other biomaterials, such as bone grafts and membranes [51].

## 3. Three-Dimensional Hydrogels as a Novel Treatment

**Figure 4:**
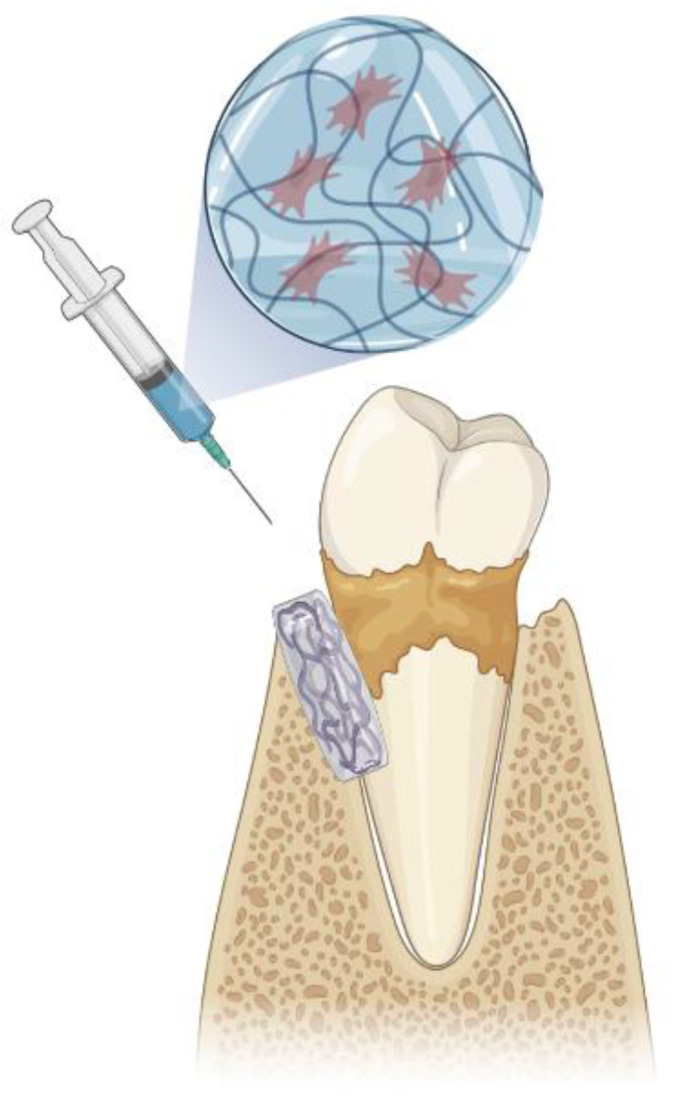
Injectable hydrogels for periodontal regeneration. Bioactive cues, such as cells, can be incorporated into the hydrogel to stimulate new tissue formation. Figure created using Biorender.com.

The ultimate goal of periodontal therapy is to achieve the regeneration of the alveolar bone, cementum, gingiva, and periodontal ligament. Thus, tissue engineering approaches have been evolving from simple membranes or grafts to more complex constructs. Hydrogels have shown interesting results for periodontal regeneration [29], since they can address one of the major limitations of current treatments: the inability to exert spatiotemporal control over the wound healing process [52]. In fact, hydrogels possess great advantages from a clinical perspective, such as injectability, easy accessibility, and their potential to deliver the necessary cues to induce the migration of the host cells and to accelerate periodontal tissue formation (Figure 4).

In addition to biochemical cues, the mechanical properties and biodegradability of hydrogels are also important. In fact, the stiffness of the material needs to be similar to the tissue’s stiffness in order to promote successful adaption of the material to the root surface. Additionally, hydrogels must degrade within a period of time similar to the growth of new tissue [53].

### 3.1. Hydrogel Composition

Hydrogels can be composed of several materials and combined with bioactive molecules to induce new tissue formation [53] (Table 3 and Table 4). Hydrogels are mainly classified into natural and synthetic polymers.

#### 3.1.1. Natural Polymers

Hydrogels composed of natural polymers are derived from natural sources. Natural polymers exhibit remarkable biocompatibility and biodegradability. Their hydrophilic nature promotes cell adhesion, proliferation, and differentiation. However, the mechanical strength and stability of natural polymers are not as high as those of synthetic hydrogels, which can limit their applications [19,20].

##### Chitosan

In particular, chitosan hydrogels have been used for periodontal regeneration, either alone or in combination with other polymers or bioactive agents. Palma and colleagues have used chitosan hydrogels in dog teeth affected with periodontitis; however, the results showed no improvement in the formation of new mineralized tissues [54]. Recent studies have shown that chitosan combined with biologic agents, such as BMP and PDLSC, showed signs of periodontal regeneration [55,56]. In vivo studies in dog furcation defects have shown beneficial outcomes when using chitosan scaffolds loaded with β-glycerolphosphate (β-GP) [57] and BMP-7 [58]. Furthermore, chitosan can also be combined with natural polymers, such as alginate, hyaluronic acid, collagen, gelatin, and carboxymethyl cellulose. Synthetic materials have also been combined with chitosan, such as β-TCP, β-GP, polyvinylpyrrolidone (PVP), PVA, and PEG [55,57,58,59,60,61,62,63,64,65]. Interestingly, most of these composite scaffolds were also combined with biologic agents, such as VEGF [61], BMP [55,58], and freeze-dried platelet concentrate (FDPC) [64].

##### Collagen

Collagen hydrogels have also been extensively studied for periodontal regeneration. Although collagen hydrogels have been mostly used as membranes for GTR, studies have evaluated their influence on periodontal regeneration when applied directly to the defect. In fact, Sato and colleagues have demonstrated that the application of a collagen gel loaded with FGF-2 in a dog defect model yielded promising results, with the formation of new collagen fibers and cementum [66].

##### Gelatin Methacrylate

Gelatin methacrylate (GelMA) hydrogels are also frequently used for periodontal regeneration. Pan and colleagues evaluated the effect of a GelMA hydrogel with embedded human PDLSC both in vivo and in vitro and observed enhanced proliferation and differentiation of PDLSC within the hydrogel, as well as newly bone formation when the hydrogels were placed on rat alveolar defects [67]. GelMA hydrogels have also been enriched with nanohydroxyapatite [68]. These constructs enhanced osteogenic differentiation of human PDLSC, and in vivo studies in a mouse model showed increased formation of mineralized tissue [68]. Apart from being used alone, GelMA has also been combined with other materials, such as polyethylene glycol diacrylate (PEGDA) [69].

##### Hyaluronic Acid

Being part of the natural ECM, hyaluronic acid has also been used for periodontal regeneration [70]. Fawzy El-Sayed and colleagues studied the effect of a hyaluronic acid hydrogel loaded with interleukin 1 receptor antagonist (IL-1ra) and gingival MSC in a swine periodontal defect model [71]. Both IL-1ra-loaded and unloaded constructs proved its potential for periodontal regeneration, yielding a higher clinical attachment level, probing depth, periodontal attachment level, cementum regeneration, and bone regeneration in addition to a lower junctional epithelium [71].

##### Self-Assembling Peptides

Recently, self-assembling peptide (SAP) hydrogels have also been investigated for periodontal repair [72,73,74]. Overall, SAP used in rat periodontal defects showed greater organization of periodontal fibers in the defect, as well as decreased epithelial downgrowth [72] and enhanced new bone formation [74].

#### 3.1.2. Synthetic Polymers

In addition to natural hydrogels, synthetic hydrogels, such as PEG and PEGDA hydrogels have also been explored [75]. Liu et al. developed a PEGDA hydrogel combined with stromal cell-derived factor 1 (SDF-1) and showed that this hydrogel promoted proliferation, migration, and osteogenic differentiation of PDLSC, and it promoted osteogenesis when used in a rat periodontitis model [76]. Tanongpitchayes and colleagues have developed a combination of a polyacrylamide-based hydrogel and nanohydroxyapatite [77]. This hydrogel effectively enhanced pocket regeneration in dogs with periodontitis.

**Table 3:** In vitro studies using hydrogels for periodontal regeneration.

Reference	Materials	Control	Cells	Main Results
Zang, S. et al., 2014 [57]	Chitosan, β-GP	Cell culture surface	Human PDLSC	No obvious cytotoxicity on human PDLSC was reported.
Xu, X. et al., 2019 [59]	Chitosan, β-GP, gelatin, aspirin, erythropoietin (EPO)	Hydrogel without aspirin and EPO	Rat bone marrow MSC	The hydrogels exhibited no toxicity in vitro. Aspirin and EPO could be continuously released from the hydrogels for at least 21 days.
Arpornmaeklong, P. et al., 2021 [60]	Chitosan, β-GP, collagen, quercetin	Hydrogel without quercetin	Human PDLSC	The quercetin hydrogels exhibited an antioxidant capacity and enhanced the growth of human PDLSC with an increasing quercetin dose.
Divband, B. et al., 2021 [61]	Chitosan biguanidine, carboxymethylcellulose, BMP-2, VEGF	Cell culture surface	Human dental pulp stem cells (DPSC)	Hydrogels were non-toxic and significantly increased DPSC proliferation. Hydrogels with BMP-2 and VEGF showed significantly higher gene and protein expression of alkaline phosphatase (ALP), Collagen I, and osteocalcin (OC); increased ALP activity and calcium deposition.
Malik, M. et al., 2020 [62]	Chitosan, carboxymethyl cellulose, nanohydroxyapatite (nHAP), thyroxine	Cell culture surface	Mouse pre-osteoblast cells (MC3T3-E1)	Hydrogels were non-toxic in vitro.
Miranda, D. et al., 2016 [63]	Chitosan, hyaluronic acid (HA)	Cell culture surface	Mouse NIH3T3 and human MG63 cell lines	No significant differences in cell viability were seen between the various formulations of the hydrogels. MG63 proliferated on all the hydrogels, observed through increasing cell viability over time.
Ammar, M. et al., 2018 [64]	Chitosan, β-GP, FDPC	Hydrogel without FDPC	Human PDLSC	All FDPC-loaded hydrogels exhibited sustained release of TGF-β1 and PDGF for two weeks. The loading of 10 and 15 mg/mL of FDPC in the hydrogels significantly increased the PDLSC viability.
Zhang, Y. et al., 2021 [65]	PEG, chitosan, acetylsalicylic acid (ASA), PDLSC	Cell culture surface	Human PDLSC	Hydrogels with ASA enhanced cell proliferation and osteogenic differentiation, observed through a significant increase in ALP activity, calcium deposition, and in RUNX2, ALP, and OC gene expression.
Fraser, D. et al., 2021 [75]	PEG, PEG-dithiol or matrix metalloproteinase (MMP)-degradable peptide crosslinker, arginylglycylaspartic acid (RGD)	Non-degradable hydrogels	Human PDLSC	MMP-degradable hydrogels with RGD significantly enhanced cell proliferation. MMP-degradable hydrogels showed a significant upregulation of periodontal genes and an increase in ALP activity compared to non-degradable hydrogels crosslinked with PEG-dithiol.
Sowmya, S. et al., 2017 [78]	Chitin, poly(lactic-co-glycolic acid) (PLGA), nanobioglass ceramic (nBGC), cementum protein 1 (CP-1), PRP, FGF-2	Hydrogels without additives	Human dental follicle stem cells	The incorporation of the additives nBGC, CP-1, PRP, and FGF-2 resulted in improved cementogenic, osteogenic, and fibrogenic differentiation of human dental follicle stem cells, similar to hydrogels without additives in induction media.
Pan, J. et al., 2020 [67]	GelMA, PDLSC	Cell culture surface	Human PDLSC	PDLSC proliferated at a similar rate in the hydrogels and in 2D culture.
Chen, X. et al., 2016 [68]	GelMA, nHAP	Hydrogels without nHAP	Human PDLSC	GelMA hydrogels with 2% nHAP enhanced the osteogenic differentiation of PDLSC by increasing ALP, OC, and RUNX2 gene expression.
Ma, Y. et al., 2017 [69]	GelMA, PEGDA, PDLSC	GelMA hydrogel	Rat PDLSC	Hydrogels with a higher GelMA concentration resulted in increased ALP activity, calcium deposition, and osteogenic gene expression by PDLSC.
Liu, S. et al., 2021 [76]	PEGDA, dithiothreitol, functional peptide module, SDF-1	Cell culture surface	Human PDLSC	Hydrogels with SDF-1 significantly enhanced cell proliferation and increased ALP activity and calcium deposition. The hydrogels strongly inhibited the growth of *Porphyromonas gingivalis.*
Koch, F. et al., 2018 [73]	11-amino acid SAP (P11-4, P11-8, P11-13, P11-14, P11-28 and P11-29)	Cell culture surface	Human PDLSC and human calvarial osteoblasts	P11-4 and P11-8 hydrogels showed higher metabolic activity than the hydrogels with the other P11 SAP and resulted in increased ALP activity and calcium deposition compared to tissue culture polystyrene (TCP).
Takeuchi, T. et al., 2016 [74]	SAP hydrogel RADA16 from PuraMatrix™	Matrigel	Rat PDLSC	RADA16 showed a significant increase in cell proliferation compared to Matrigel.
Koch, F. et al., 2020 [79]	P11-4 SAP or Collagen I	Cell culture surface	Human PDLSC	P11-4 hydrogels and TCP showed similar metabolic activity, which was higher than in collagen hydrogels. Cells migrated and deposited ECM proteins in the P11-4 hydrogels. Collagen I expression was higher in the P11-4 hydrogels than in TCP after 7 days.
Yoshida, W. et al., 2019 [80]	SAP gel SPG-178 from PanaceaGel^®^	Cell culture surface	Rat PDLSC	1.5% SPG-178 showed significantly higher cells viability/proliferation than 0.8% SPG-178.
Babo, P. et al., 2017 [81]	Methacrylated hyaluronic acid, platelet lysate (PL)	Cell culture surface	Human PDLSC	PL release provided antimicrobial action to the hydrogels, increasing with higher PL content. Hydrogels with higher amount of PL showed increased metabolic activity of cells encapsulated or seeded in the hydrogels.
Tan, J. et al., 2019 [82]	Nap-Phe-Phe-Tyr-OH (NapFFY), SDF-1, BMP-2	Cell culture surface	Rat bone MSC (BMSC)	All hydrogels were not cytotoxic and stimulated cell proliferation. SDF-1 and BMP-2 significantly increased ALP gene expression.
Xu, Y. et al., 2020 [83]	Sodium alginate (SA), cubic cuprous oxide, polydopamine-coated titanium dioxide	Cell culture surface	BMSC, human umbilical vein endothelial cells, human fibroblasts	Hydrogels doped with the additives (CTP-SA) showed increased antibacterial activity. CTP-SA hydrogels irradiated with dual light (blue and near-infrared) resulted in a significant increase in ALP activity and in OC and RUNX2 gene expression by BMSC.
Juriga, D. et al. 2022 [84]	Polyaspartic acid, dopamine (DA)	Hydrogel without DA	Human PDLSC and SH-SY5Y human neuroblastoma cell line	Hydrogels with DA at a higher concentration showed lower cell viability. Both cell types proliferated and migrated in the hydrogels. Hydrogels with a DA concentration of 1/20 showed vertical cell penetration from the top of the hydrogel in the depth, with PDLSC having a slightly higher migration potential.

**Table 4:** In vivo studies on hydrogels for periodontal regeneration.

Reference	Materials	Control	Model	Main Results
Palma, P. et al., 2017 [54]	Chitosan, sodium hyaluronate, or pectin	Autologous blood clot	Immature dog teeth with apical periodontitis	The incorporation of chitosan hydrogels in dogs did not improve the formation of new mineralized tissues along the root canal walls or the histologic evidence of the regeneration of a pulp-dentin complex. Moreover, they demonstrated optimal properties for bone tissue engineering applications.
Chien, K. et al., 2018 [55]	Chitosan, gelatin, GP, BMP-6, induced pluripotent stem cells (iPSC)	Hydrogel without BMP-6 and iPSC	Rat periodontal defect model	Synergistic effects of iPSC and BMP-6 increased both bone and cementum formation. BMP-6/iPSC-loaded hydrogels showed reduced levels of inflammatory cytokines, new periodontal ligament formation, and new bone synthesis, observed through a significantly higher bone volume fraction and trabecular number.
Yan, X. et al., 2015 [56]	Chitosan, PDLSC	Untreated defect	Rat intrabony periodontal defect	Gels with and without cell loading showed no differences in periodontal regeneration. All hydrogels were biodegradable and improved periodontal regeneration in terms of functional ligament length.
Zang, S. et al., 2014 [57]	Chitosan, β-GP	Untreated defect	Dog class III furcation defect	These hydrogels promoted periodontal tissue regeneration, observed through significantly increased new bone and cementum formation.
Zang, S. et al., 2019 [58]	Chitosan, β-GP, BMP-7, ornidazole (ORN)	Sham surgery	Beagle dog class III furcation defect model	Hydrogels loaded with ORN exhibited antimicrobial activity against *P. gingivalis*. Defects treated with BMP-7 loaded hydrogels showed significantly more new bone and cementum and less connective tissue.
Xu, X. et al., 2019 [59]	Chitosan, β-GP, gelatin, aspirin, EPO	Untreated defect	Rat periodontitis model	Hydrogels exhibited no toxicity in vivo. Aspirin/EPO-loaded hydrogels showed significant anti-inflammatory effects and resulted in improved bone regeneration with significantly higher bone volume/tissue volume and bone mineral density.
Malik, M. et al., 2020 [62]	Chitosan, carboxymethyl cellulose, nHAP, thyroxine	Hydrogel without thyroxine	Fertilized chicken eggs	Hydrogels containing a lower amount of thyroxine showed maximum neovascularization, as assessed through the chick chorioallantoic membrane assay. Blood vessels penetrated all thyroxine-loaded hydrogels.
Zhang, Y. et al., 2021 [65]	PEG, chitosan, ASA, PDLSC	Untreated defect	Mouse calvarial bone defect	ASA significantly improved bone regeneration, and PDLSC-laden hydrogels with ASA resulted in the highest amount of newly formed bone.
Abdelrasoul, M. et al., 2022 [85]	Alginate, chitosan, β-TCP, melatonin	Sham surgery	Mongrel dogs class II furcation defect model	Melatonin-loaded hydrogels accelerated the formation of new bone and enhanced the quality of newly formed bone, allowing complete periodontal regeneration. The scaffold prevented overgrowth and entrapment of epithelial cells in furcation defects.
Xu, Y. et al., 2020 [83]	SA, cubic cuprous oxide, polydopamine-coated titanium dioxide	Untreated defect	Mouse periodontal bone defect	CTP-SA hydrogels irradiated with dual light (blue and near-infrared) resulted in significantly improved bone regeneration in vivo. CTP-SA hydrogels irradiated with blue light showed significantly less inflammatory cells and decreased Tumour necrosis factor (TNF- α) expression.
Sato, Y. et al., 2004 [66]	Collagen, FGF-2	Hydrogel without FGF-2	Beagle dog cementum defect of the root surface	At 4 weeks post-surgery, random periodontal ligament fibers were bound to dentin. At 8 weeks post-surgery, the use of hydrogels with FGF-2 resulted in the formation of dense fibers bound to alveolar bone and significantly more newly synthesized cementum.
Pan, J. et al., 2020 [67]	GelMA, PDLSC	Untreated defect	Rat alveolar bone defect	Hydrogels with PDLSC showed significantly improved bone regeneration at 4 and 8 weeks post-surgery compared to pure GelMA hydrogels.
Chen, X. et al., 2016 [68]	GelMA, nHAP	Hydrogel without nHAP	Subcutaneous implantation in nude mice	Hydrogels with 2% nHAP increased mineralized tissue formation with abundant vascularization compared to hydrogels with 0%, 1%, and 3% nHAP.
Ma, Y. et al., 2017 [69]	GelMA, PEGDA, PDLSC	Saline and GelMA/PEGDA hydrogel	Rat alveolar bone defect model	PDLSC-laden hydrogels significantly increased new bone formation in the defects.
Liu, S. et al., 2021 [76]	PEGDA, dithiothreitol, functional peptide module, SDF-1	Untreated defect	Rat periodontitis model	SDF-1 loaded hydrogels showed improved bone regeneration, decreased TNF-α and IL-1β expression, and facilitated the recruitment of CD90^+^/CD34^−^ stromal cells.
Sowmya, S. et al., 2017 [78]	Chitin, PLGA, nBGC, CP-1, PRP	Sham surgery	Rabbit maxillary periodontal defect model	Complete defect closure and healing; formation of new cementum, fibrous periodontal ligament, and alveolar bone with well-defined bony trabeculae.
Kinoshita, A. et al., 1997 [86]	Gelatin, PLGA, BMP-2	Hydrogel without BMP-2	Beagle dog premolar furcation defect model	BMP-2 hydrogels showed apparent periodontal tissue regeneration occupying the majority of the defects and resulted in a significantly greater amount of new bone and cementum.
Shen, S. et al., 2021 [87]	HA, PLGA microspheres, 6-Bromoindir- ubin-3′-oxime (BIO)	Untreated defect	Mouse periodontitis model	HA-PLGA-BIO hydrogels significantly enhanced bone regeneration and the expression of osteogenic markers ALP, RUNX2, and OC, resulting in lower infiltration of inflammatory cells.
Fawzy El-Sayed, K. et al., 2015 [71]	HA, gelatin, gingival MSC, interleukin-1 receptor antagonist (IL-1ra)	Untreated defect	Miniature swine periodontal defect model	Cell-laden hydrogels with and without IL-1ra resulted in a significantly higher periodontal attachment level and clinical attachment level, reduced junctional epithelium length, improved bleeding on probing, and increased cementum and bone regeneration.
El-Sayed, B. et al., 2020 [72]	P11-4 SAP	Untreated defect	Rat periodontal defect model	Defects treated using P11-4 hydrogels showed a greater organization of periodontal fibers, a significant increase in functional periodontal ligament length, and a reduction in epithelial down growth.
Takeuchi, T. et al., 2016 [74]	SAP hydrogel RADA16 from PuraMatrix™	Untreated defect	Rat periodontal defect model	RADA16 resulted in significantly increased bone volume, trabecular thickness, and reduced trabecular separation compared to Matrigel-treated and untreated defects. The angulation of fiber bundles was also significantly greater with RADA16 and closer to the natural periodontal ligament.
Yoshida, W. et al., 2019 [80]	SAP gel SPG-178 from PanaceaGel^®^	Untreated defect	Rat periodontal defect	Parathyroid hormone used as an adjuvant with SPG-178 improved bone regeneration and resulted in greater fiber angulation similar to natural periodontal ligament and an increased number of VEGF- and OSX-positive cells.
Tan, J. et al., 2019 [82]	NapFFY, SDF-1, BMP-2	Untreated defect	Rat maxillary critical-sized periodontal bone defect	Synergistic effects of SDF-1 and BMP-2 were observed. The NapFFY/SDF-1/BMP-2 hydrogel showed a significant increase in bone regeneration compared to NapFFY, NapFFY/SDF-1, and NapFFY/BMP-2.
Tanongpitchayes, K. et al. 2021 [77]	Polyacrylamide, nHAP	No control	Dog periodontitis model	Radiographic grading, alveolar bone height, and intensity continuously showed significant increases in the weeks following the treatment of post-extraction sockets using the hydrogels.
Struillou, X. et al., 2011 [88]	Hydroxypropyl methyl cellulose, biphasic calcium phosphate (BCP)	Untreated defect	Canine fenestration and premolar furcation defects	BCP granules were retained in the defect during the healing phase. The hydrogels promoted new bone formation 3 months after implantation in the defects.

Abbreviations in Table 3 and Table 4 in order of appearance: Materials: β-GP: β-glycerol phosphate, EPO: Erythropoitin, BMP-2: bone morphogenetic protein-2, VEGF: vascular endothelial growth factor, nHAP: nanohydroxyapatite, HA: hyaluronic acid, FDPC: freeze-dried platelet concentrate, PEG: polyethylene glycol, ASA: acetylsalicylic acid, PDLSC: periodontal ligament stem/stromal cells, MMP: matrix metalloproteinase, RGD: arginylglycylaspartic acid, PLGA: Poly(Lactic-co-Glycolic Acid), nBGC: nanobioglass ceramic, CP-1: Cementum protein-1, PRP: platelet-rich plasma, FGF-2: fibroblast growth factor-2, GelMA: gelatin methacrylate, PEGDA: poly(ethylene glycol) diacrylate, SDF-1: stromal cell-derived factor-1, SAP: self-assembling peptide, PL: platelet lysate, NapFFY: Nap-Phe-Phe-Tyr-OH, SA: sodium alginate, DA: dopamine, GP: glycerol phosphate, iPSC: induced pluripotent stem cells, ORN: ornidazole, β-TCP: β-tricalcium phosphate, BIO: 6-bromoindir-ubin-3′-oxime, MSC: mesenchymal stromal cells, IL-1ra: interleukin-1 receptor antagonist, BCP: biphasic calcium phosphate; Cells: DPSC: dental pulp stem cells, BMSC: bone-derived MSC; Main Results: ALP: alkaline phosphatase, OC: osteocalcin, TGF-β1: transforming growth factor β1, PDGF: platelet-derived growth factor, RUNX2: Runt-related transcription factor 2, TCP: tissue culture polystyrene, TNF-α: tumor necrosis factor alpha, IL-1β: interleukin-1 beta, OSX: Osterix.

### 3.2. Methods of Fabrication

Hydrogels can be fabricated by crosslinking networks and can be classified as physically or chemically crosslinked hydrogels [89,90]. Physical crosslinking is typically accomplished through physical mechanisms, including crystallite formation, polymer chain complexion, hydrophobic interaction, and the establishment of hydrogen bonds [91]. Chemical or covalent crosslinking results from covalent bond junctions. Physically crosslinked hydrogels exhibit reversibility due to conformational changes that prevent dissolution in aqueous media, while chemically crosslinked hydrogels are permanent and irreversible owing to configurational changes. The macroscopic properties of hydrogels, such as the degree of swelling, mechanical characteristics, and the transport of molecules through the hydrogel meshes, are impacted by both the type and degree of crosslinking [92].

In the field of tissue engineering, hydrogels have traditionally presented limited mechanical strength and structural complexity. However, due to their expanding applications, there is a growing need for advanced engineering methods that allow for precise control of both the physical and chemical properties of hydrogels, enabling the creation of more well-defined structures. Recent advanced engineering methods, including 3D bioprinting and in situ gel formation, have been developed for fabricating hydrogels for periodontal applications.

#### 3.2.1. Three-Dimensional Bioprinting

Three-dimensional bioprinters can create customized scaffolds that closely mimic the natural architecture of periodontal tissues [93]. Bioprinters can deposit cells, biomaterials, and bioactive molecules with high precision, allowing for the creation of complex periodontal structures and ensuring that the right cell types are in the right locations. Furthermore, 3D bioprinting enables the incorporation of PDLSC into the scaffold, promoting tissue regeneration by differentiating into the necessary cell types. Regarding drug and growth factors delivery, bioprinters can precisely control the release of growth factors or antimicrobial agents, which is essential for managing inflammation, promoting tissue regeneration, and preventing infections in periodontal applications.

A recent study from Miao and colleagues developed a 3D bioprinted multi-component hydrogel for cell delivery in periodontal tissue regeneration [93]. The hydrogel consisted of GelMA, sodium alginate, and a bioactive glass microsphere. Furthermore, this hydrogel was used as a bioink to load mouse bone marrow MSC and growth factors (BMP-2 and PDGF) to develop scaffolds for periodontal applications. The cells loaded in the hydrogel maintained good cellular viability after 3D bioprinting and presented enhanced osteogenic differentiation in BMP-2- and PDGF-loaded hydrogels. Moreover, when the hydrogels were transplanted in beagle dog periodontal defects, a significant regeneration of gingival tissue, periodontal ligament, and alveolar bone was observed [93].

In a different study from Yan et al., a 3D bioprinted periodontal construct was developed with high architectural integrity using a GelMA/decellularized ECM cell-laden bioink [94]. Dental follicle cells were encapsulated into the bioink. After incorporation of the 3D bioprinted constructs into a critical-size periodontal defect model, enhancement of the regeneration of periodontal tissues in beagles was observed. In particular, anchoring structures of the bone–ligament interface, well-aligned periodontal fibers, and highly mineralized alveolar bone were observed [94].

#### 3.2.2. In Situ Gel Formation

Regarding periodontal applications, in situ gel formation involves the use of a gel-like material that can be administered directly at the defect site and transformed into a solid state once it comes into contact with oral tissues. The formation of in situ gels is generally a time-dependent process and can vary depending on the specific formulation [95]. The gel formation process may involve factors including temperature, pH, or the presence of specific ions or polymers [95].

In situ gel-forming systems have the ability of sustained drug release and have attracted attention due to their easy administration (injectable) and high drug retention (localization) in periodontal defects.

Recently, Gopalakrishna and colleagues developed a piperine-loaded in situ gel [96]. Different gel formulations were tested by varying the concentration of deacylated gellan gum crosslinked with sodium tripolyphosphate and poloxamer-407. The optimized formula was implanted into human patients for 14 days, and its anti-inflammatory effectiveness was evaluated. At physiological conditions, the hydrogel was able to form, allowing an efficient residence time of the hydrogel within the defect. Furthermore, it was possible to observe a significant reduction in the mean plaque score, gingival index and pocket depth, and anti-inflammatory potential compared to the control group [96].

In a different work, Swain et al. developed a moxifloxacin hydrochloride-loaded in situ gel for the treatment of periodontitis [97]. Different formulations were tested by varying temperature sensitive (poloxamer 407), ion sensitive (gellan gum), and pH sensitive (carbopolol 934P) polymers. The optimized formulation contained 19.072% *w*/*v* poloxamer 407 and 0.245% *w*/*v* gellan gum, which has a desired gel temperature of 36 °C and gelling time of 102 s, and 98% of the drug released after 9 h [97].

Ranch and colleagues developed a doxycycline hyclate-laden in situ gel composed of poloxamer 407, chitosan, and polytethylene glycol 600 [98]. After testing, the gelation temperature of the optimized in situ gel was 34 ± 1 °C with a sufficient strength and texture profile for periodontal applications. Furthermore, the in vitro dug release assays demonstrated a sustained release from the developed gels (24 h) compared to commercially available gels (7 h). Interestingly, doxycycline hyclate retained its antimicrobial efficacy when formulated as an in situ gelling system [98].

### 3.3. Clinical Studies on the Application of Hydrogels for Periodontal Repair

Clinical studies on hydrogels for periodontal applications have been investigated [99]. Hyaluronic acid hydrogels loaded with human fibroblast growth factor 2 were used to treat periodontal defects [20]. After 1 year of treatment, the clinical parameters of periodontal wound healing were significantly improved in a total of 30 patients [20].

Olszewska-Czyz and colleagues have shown that the use of hyaluronic acid hydrogels as an adjunctive to non-surgical periodontal therapy demonstrated more favorable clinical results after 3 months [100]. A reduction in inflammation was observed, measured by bleeding on probing (−6% compared to the control group) and gain in periodontal attachment (1 mm more than control group), while it had no effect on the probing depth reduction. Furthermore, no side effects were reported [100].

In a different study, Tamura et al. reported the clinical effects of the sustained release of basic fibroblast growth factor (bFGF) from gelatin hydrogels in patients with periodontal disease presenting bone defects [101]. A total of 23 patients were treated with a mucoperiosteal flap operation. At the time of surgery, each bone defect was filled with a bFGF-gelatin hydrogel. One year after the treatment, there were significant improvements in clinical parameters, such as probing pocket depth reduction, clinical attachment gain, and radiographic bone fill. Furthermore, no adverse effects were observed [101].

Gad and colleagues formulated solid lipid microparticles gels encapsulating doxycycline hydrochloride and metronidazole and proved the clinical efficacy of these gels in periodontal patients [102].

## 4. Conclusions and Future Perspectives

Current clinical approaches for periodontal regeneration are focused on the use of membranes and bone grafts. Recently, these materials have been used for the delivery of growth factors and bioactive molecules to promote and enhance the wound healing process and further periodontal tissue formation. However, these strategies fail to yield the expected clinical outcomes since they do not promote the regeneration of all the periodontal tissues, including hard tissues (cementum and alveolar bone) and soft tissues (gingiva, periodontal ligament). Thus, hydrogels have been used in several applications in the field of tissue engineering, such as space fillers, vehicles for delivery of bioactive molecules, and as 3D structures to promote new tissue formation [29,52,53].

Periodontal tissue regeneration is a very complex process. Hydrogels have been explored as scaffolds and/or drug delivery systems due to their capacity to incorporate cells into their structures and their ability to degrade on the same timeline as new tissue formation. Additionally, hydrogels can be combined with several bioactive molecules to induce cellular processes, such as migration, proliferation, differentiation, vascularization, and mineralization [96,97,98]. Furthermore, hydrogels have excellent biocompatibility, water retention, and controlled release, and they provide support for cellular interaction during periodontal regeneration [15,16]. Regarding periodontal applications, since hydrogels are structurally similar to the ECM of several tissues and may be delivered in a minimally invasive manner, they can reduce the inflammatory response to remodel the structure and function of periodontal tissues. In fact, as a mechanism of action, the sustained release of drugs presented by hydrogels allows for the control of disease by reducing inflammation and attracting host cells to the defect site. Several therapeutic agents, such as antibiotics, anti-inflammatory and osteogenic drugs, have been loaded into hydrogels, presenting effective benefits in periodontal health and allowing for sustained drug release [103]. In fact, antimicrobial hydrogels are an attractive solution to control infectious diseases and to address the challenges associated with antibiotic resistance due to their unique physicochemical and biological properties and drug delivery capacity. Moreover, combining complementary therapeutic approaches with these antimicrobial hydrogels will improve their effectiveness. These hydrogels can have inherent antimicrobial activities or can be loaded with antimicrobial agents [104,105].

Although there are only a few clinical studies related to the effectiveness of hydrogels for periodontal therapy, some clinical studies have already shown promising results on the application of hydrogels for periodontal regeneration [20,100,101,102]. Several hydrogels loaded with different bioactive agents have shown significant improvements in clinical parameters, such as probing pocket depth reduction, clinical attachment gain, and radiographic bone fill. Although remarkable progress has been made in the application of hydrogels in periodontal regeneration, challenges remain to providing sufficient mechanical strength and more biological properties to these hydrogels to achieve successful regenerative outcomes. In fact, the composition and structure of hydrogels have a significant impact on periodontal tissue regeneration. ECM-derived scaffolds prepared from decellularized tissues or cell cultures have been developed to promote functional tissue remodeling in several clinical applications. Indeed, the decellularized ECM can be manipulated to form hydrogels that can be used as injectable materials to fill irregularly shaped defects. By having ECM structural and biological cues, these hydrogels direct cell behavior and influence new tissue formation. Furthermore, the development of intelligent and multifunctional hydrogels for periodontal tissue regeneration is required for future research. While 3D bioprinting holds significant promise for periodontal applications, it is important to note that the technology is still evolving, and clinical translation is ongoing. Researchers and clinicians are actively working to optimize bioprinting techniques for better outcomes in the treatment of periodontal disease. Additionally, regulatory and safety considerations are crucial when moving from lab research to clinical practice. Further investigation is needed to determine which combinations of biomolecules, cells, and hydrogels can improve clinical results. This research will also require extensive teamwork between clinicians and researchers. This paper reviews the current treatments for periodontal disease and new insights on hydrogels for periodontal regeneration, providing discussions about their different characteristics and outcomes and aiming to contribute to successful periodontal regeneration.

## Figures and Tables

**Table 1:** Cell types and molecules responsible for periodontal regeneration.

Component	Type of Component	Examples
Cellular	Fibroblasts	Gingival fibroblasts, periodontal ligament fibroblasts
	Osteogenic cells	Osteoblasts, osteoclasts, cementoblasts
	Immune cells	Neutrophils, macrophages, lymphocytes
Molecular	Growth factors (GF)	Bone morphogenetic proteins, chlorella GF, epidermal GF, fibroblast GF, insulin-like GF, platelet-derived GF
	ECM proteins	Bone sialoprotein, collagen, enamel matrix proteins, fibronectin, hyaluron, laminin, osteocalcin, osteopontin, proteoglycans, osteonectin, tenascin

**Table 2:** Bone grafts and substitute materials used for periodontal applications, categorized according to their source.

Type of Graft	Material Source	Examples
Natural	Autogenous	Extra-oral: cranium, iliac crest, fibula, radius, rib, tibia. Intra-oral: anterior maxillary sinus, anterior nasal-spine, ascending ramus, coronoid process, incisive fossa, palate, torus [33].
	Allogeneic	Freeze-dried bone matrix, demineralized bone matrix [34].
	Xenogeneic	Chitosan, silk, bovine-, porcine-, and equine-derived bone substitutes [35].
	Plant-based	Gusuibu, Algae-, and coral-based bone substitutes [36].
Synthetic	Calcium phosphate	Hydroxyapatite, tricalcium phosphate, biphasic calcium phosphate, bioglass [37].
	Polymers	Polylactic acid, polyglycolic acid, polycaprolactone, polymethyl metacrylate [38].
	Metals	Nickel–titanium, magnesium [39].

## Data Availability

Not applicable.
